# Supplementary material for: Uptake of fucosylated type I human milk oligosaccharide blocks by Bifidobacterium longum subsp. infantis
Source: mBio. 2025 Jul 14;16(8):e00368-25. doi: 10.1128/mbio.00368-25 (PMC12345172; doi:10.1128/mbio.00368-25)
Supplement: Supplemental Information — Supplemental figures, tables, and molecular dynamics simulation details. [file mbio.00368-25-s0002.pdf]

Supplementary Material

Uptake of fucosylated type I human milk oligosaccharide blocks by *Bifidobacterium longum* subsp. *infantis*

Authors:

Morten Ejby Hansen[1*], Mikiyasu Sakanaka[2,4*$], Mathias Jensen[1*], Hiroka Koguchi Sakanaka[1], Michael Jakob Pichler[1], Shingo Maeda[2], Julie Franck Høvring[3], Aruto Nakajima[4], Sonja Kunstmann[1], Tine Sofie Nielsen[1], Günther Herbert Johannes Peters[3], Dirk Jan Slotboom[5], Jens Preben Morth[1], Takane Katayama[4], Maher Abou Hachem[1]

Affiliations:

[1]Department of Biotechnology and Bioengineering, Søltofts Plads, Technical University of Denmark, Lyngby DK-2800 Kgs. Denmark

[2]Faculty of Bioresources and Environmental Sciences, Ishikawa Prefectural University, Nonoichi, Ishikawa 921-8836, Japan

[3]Department of Chemistry, Kemitorvet, Technical University of Denmark, Lyngby DK-2800 Kgs. Denmark

[4]Graduate School of Biostudies, Kyoto University, Kyoto 606-8502, Japan

[5]Membrane Enzymology, Institute for Biomolecular Sciences & Biotechnology, Rijksuniversiteit Groningen, Nijenborgh 4, 9747 AG Groningen, The Netherlands

[$]Present address: Graduate School of Biostudies, Kyoto University, Kyoto 606-8502, Japan

Correspondence: maha@bio.dtu.dk (Maher Abou Hachem)

[*]Equal contribution

Supplementary Figures

FIG S1 Isothermal titration calorimetry binding analysis.

FIG S2 The topology of *Bi*GFL-BP in complex with the H1 trisaccharide.

FIG S3 Structural root square mean displacement analysis (RMSD).

FIG S4 Average RMSFs of the backbone atoms based on the triplicate simulations.

FIG S5 Comparison of ligand conformations from MD simulations and crystal structure.

FIG S6 Depletion of the Le^{b} tetrasaccharide after incubation of *B. infantis* cells.

FIG S7 Gene disruption of the two different ABC transporter SBPs in *B. infantis*.

Supplementary Tables

Table S1 Binding kinetics of *Bi*GFL-BP determined using surface plasmon resonance.

Table S2 Data collection and refinement statistics for *Bi*GFL-BP.

Table S3 Calculated free energy for ligand binding.

Table S4 The top five structural orthologs of *Bi*GFL-BP.

Table S5 Primers for cloning and mutation of *Bi*GFL-BP (Blon_0883) gene.

Supplementary methods and results

Molecular dynamic simulation details

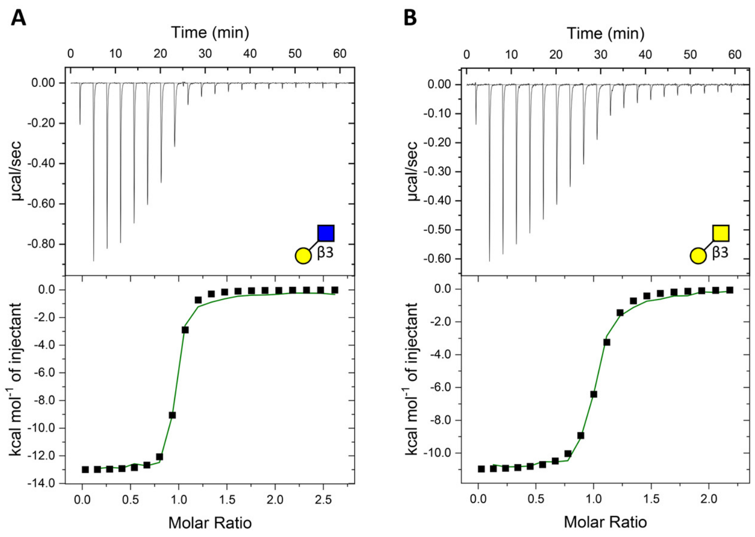

FIG S1 Isothermal titration calorimetry binding of GNB and LNB to Blon_0883 (*Bi*GFL-BP). (A) and (B) depict the binding of LNB and GNB to Blon_0883, respectively at 25 °C and pH 6.5. Upper panels show the binding thermograms and bottom panels show the binding isotherms and the fit of a one binding site model to the data (green line). The data are from a single experiment.

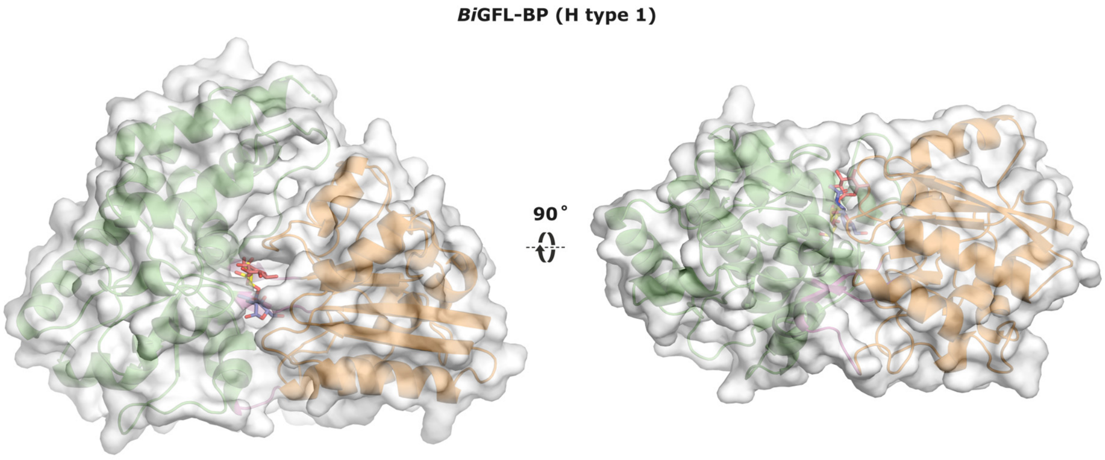

FIG S2 The topology of *Bi*GFL-BP in complex with the H1 trisaccharide. The H1 trisaccharide is bound at the interface of the two domains coloured similarly to FIG 2. The galactose (yellow) and the *N*-acetylglucosamine (blue) that form the LNB backbone are visible through the semi-transparent surface, whereas the fucosyl unit (salmon) is bound in a spacious cavity and is pointing out towards the bulk of the solvent. The left and right panels show the protein from a top and side views, respectively.

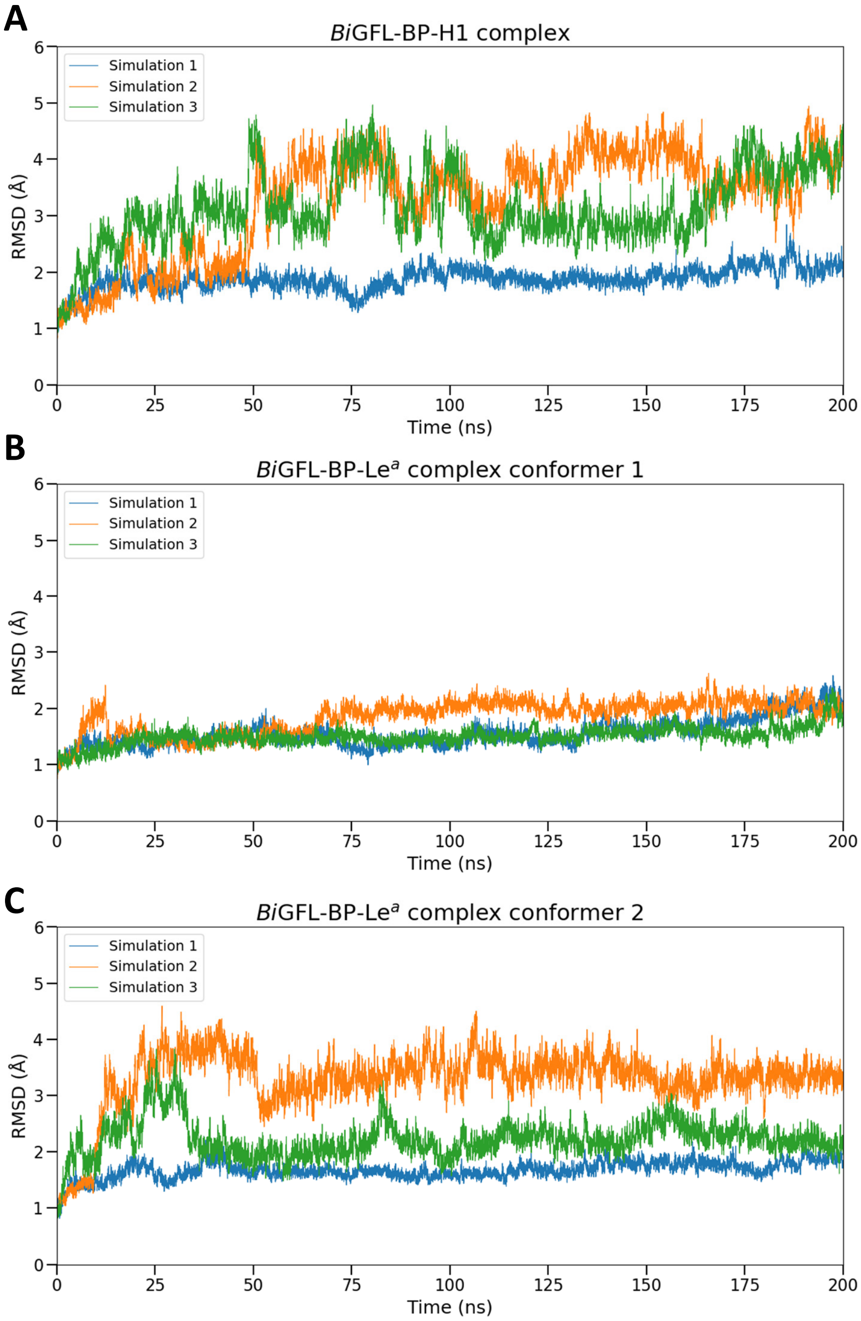

FIG S3 Structural root square mean displacement analysis (RMSD). Time course evolution of the heavy backbone RMSD of *Bi*GFL-BP in complex with (A) the H1, (B) the Le^a conformer 1 and (C) the Le^a conformer 2.

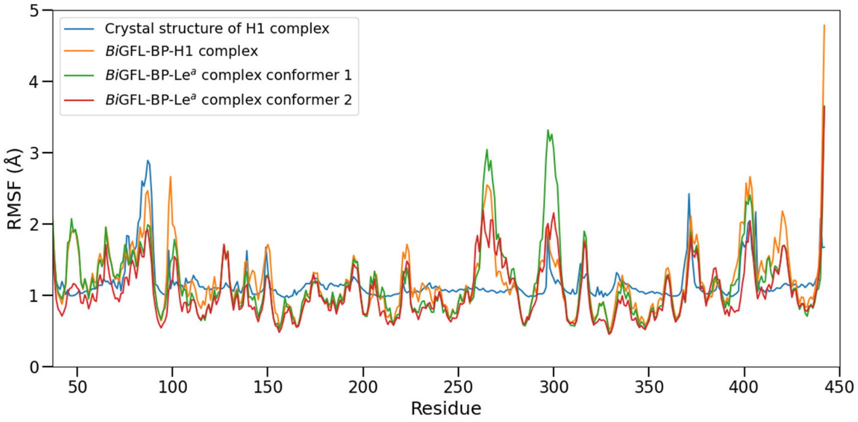

FIG S4 Average RMSFs of the backbone atoms based on the triplicate simulations.

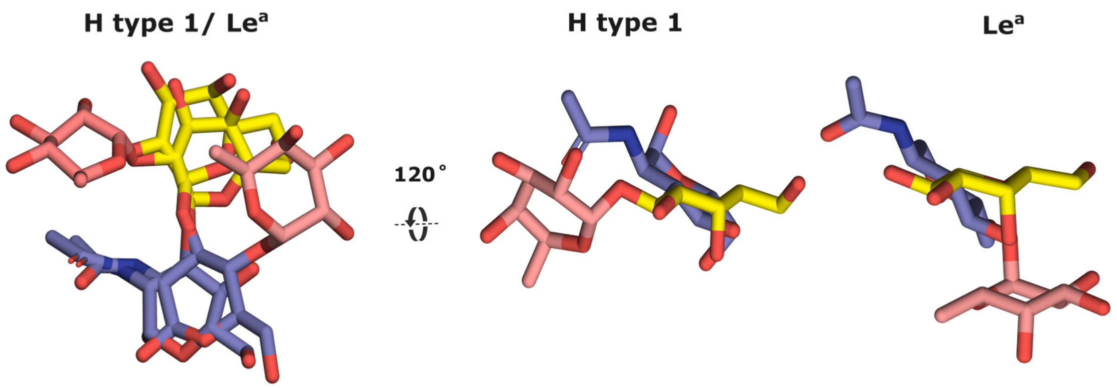

FIG S5 Comparison of ligand conformations from MD simulations and crystal structure. The left panel shows the overlay of the Le[a] trisaccharide in conformation 1 with the H1 trisaccharide from the crystal complex structure of *Bi*GFL-BP. The fucosyl unit (salmon) in the Le[a] simulated structures is bound at a less spacious cavity as opposed to the spacious and solvent connected cavity where the fucosyl unit is bound in the H1 trisaccharide complex. The LNB backbones overlay reasonably well, but are not perfectly aligned.

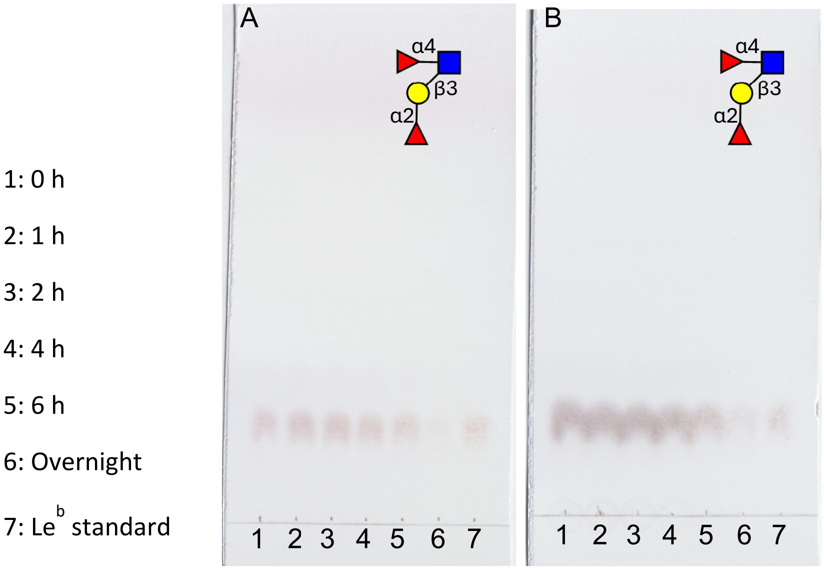

FIG S6 Depletion of the Le^b tetrasaccharide after incubation of *B. infantis* cells. *B. infantis* cells, grown on 0.5 % (w/v) complex human oligosaccharide mixtures, were washed three times and resuspended in 30 µL containing 2 mM Le^b tetrasaccharides (LNB with a double fucosylation (red triangles) to an OD_{600}=6. A) and B) represent the TLC analysis of 1 µL and 3 µL of the supernatant of the cell suspensions with the Le^b tetrasaccharides after 0, 1, 2, 4, 6 hours and overnight respectively. The depletion of this of this tetrasaccharide may be attributed to lower affinity slow uptake.

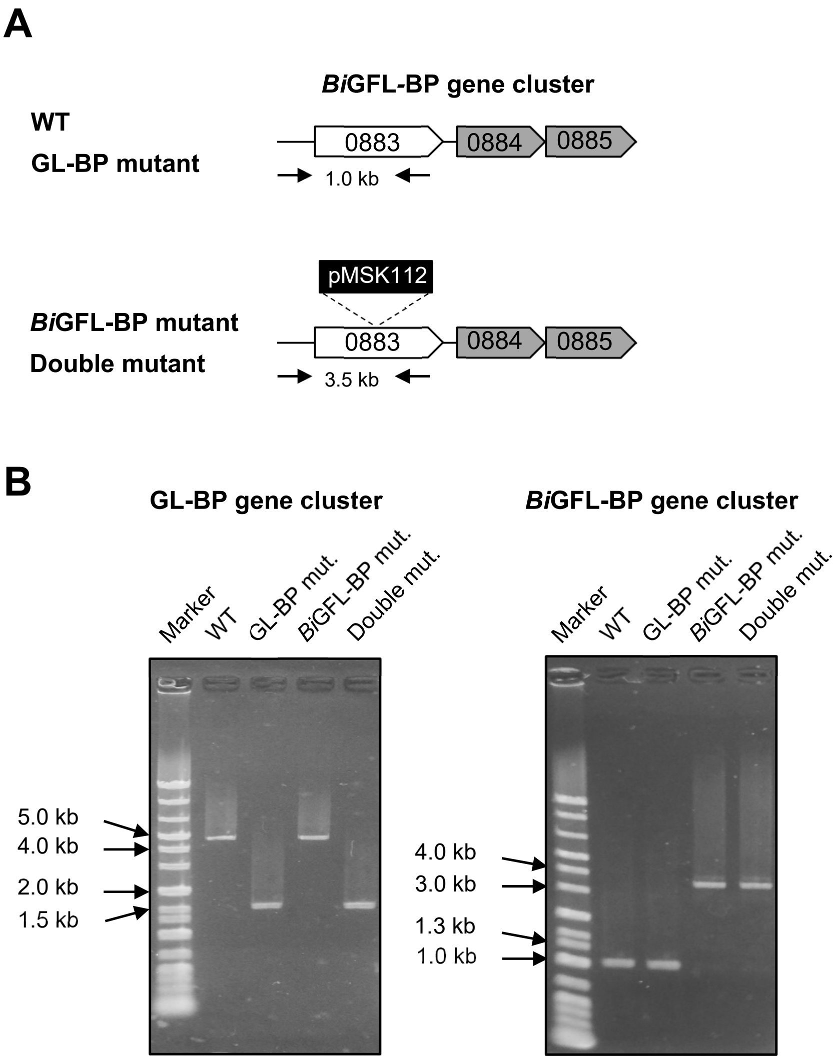

FIG S7 Gene disruption of *Bi*GFL-BP (Blon_0883) in *B. infantis* WT and GL-BP mutant. (A) Schematic representation of the *Bi*GFL-BP-associated ABC transporter gene cluster (Blon_0883–0885) in the genomes of *B. infantis* WT and derivatives. (B) Genotype analyses of each strain by agarose gel electrophoresis. The mutations introduced into the target genes were verified by PCR using genomic DNA as a template. The GL-BP mutant was generated by deleting Blon_2175–2177, while the *Bi*GFL-BP mutant was created by introducing an insertion of a suicide plasmid into the internal region of Blon_0883, *i.e.* only the binding protein was inactivated. The primers used for *Bi*GFL-BP cluster are shown in (A) with the expected sizes of the PCR products. See also Table S5.

Supplementary Tables

Table S1 Binding kinetics of *Bi*GFL-BP determined using surface plasmon resonance.

	k_{on} ($\times 10^6$ $M^{-1}s^{-1}$)	k_{off} (s^{-1})	K_d [A]Kinetics/[B]steady state(nM)		[C]R_{max} (R.U.)
Lacto-*N*-biose I	14.6 ± 0.03	0.16±0.003	11	32.5±3.2	13.2±0.32
Galacto-*N*-biose	0.85±0.004	0.04±0.003	42	62.0±4.0	14.3±0.24
Le^a	3.0±0.01	0.14±0.0005	49	146±12	16.2±0.29
H1	4.8 ± 0.06	0.17±0.002	37	158±9.0	17.1±0.23

[A]The K_d values determined from a global fit of the sensograms of five or six ligand concentrations in the range 3.42-109.4 nM.
[B]The K_d values determined from steady state fits of the sensograms from 8−10 ligand concentrations in the range 3.42nM to 2.5[illegible]M.
[B]The maximum response value based on the steady state fit
The data in all the SPR experiments are based on triplicate experiments and the data are shown as means ± standard deviations. Due to the rapid dissociation, the k_{off} is not reliably modelled and the kinetic data are not considered reliable but are included to show the kinetics signature trends.

**Table S2. Data collection and refinement statistics for *Bi*GFL-BP.**			
	**GNB**	**LNB**	**H type 1 trisaccharide**
PDB accession	9H0N	9H0O	9H0P
Resolution range (Å)	43.41 - 1.4 (1.42 - 1.4)	49.58 - 2.144 (2.2 - 2.14)	67.29 - 2.7 (2.87 - 2.7)
Space group	P 21 21 21	P 21 21 21	P 32 2 1
Unit cell (Å, °)	52.1 53.25 149.92, 90 90 90	50.07 52.56 149.319, 90 90 90	95.906 95.906 114.768, 90 90 120
Total reflections	(97476)	(45219)	563525 (93448)
Unique reflections	79467 (1956)	21409 (1112)	27544 (4517)
Completeness (%)	95.70 (67.31)	95.20 (66.78)	99.87 (99.89)
Mean I/σ(I)	34.34	10.35	5.85 (1.07)
Wilson B-factor (Å^2)	21.91	38.23	32.44
Reflections used in refinement	79467 (1956)	21409 (1112)	17217 (2831)
Reflections used for R_{free}	3890 (109)	1069 (55)	861 (141)
R_{work}	0.168 (0.444)	0.216 (0.397)	0.174 (0.215)
R_{free}	0.190 (0.418)	0.258 (0.428)	0.231 (0.302)
Number of non-hydrogen	3701	3496	3335
macromolecules	3167	3162	3153
ligands	26	42	37
solvent	508	292	145
Protein residues	408	407	406
RMS (bonds)	0.009	0.002	0.003
RMS (angles)	0.98	0.39	0.61
Ramachandran favored (%)	97.52	96.54	97.52
Ramachandran allowed (%)	1.98	3.21	1.98
Ramachandran outliers (%)	0.50	0.25	0.50
Rotamer outliers (%)	0.60	0.30	0.60
Clashscore	1.43	2.54	1.75
Average B-factor	29.09	47.17	33.06
macromolecules	27.68	47.11	33.12
ligands	18.13	47.73	33.45
solvent	38.45	47.70	31.51

**Table S3. Calculated free energy for ligand binding.**	
*Bi*GFL-BP complex	Average free binding energy (kcal mol^{-1})
*Bi*GFL-BP-H1 complex	-38±12
*Bi*GFL-BP-Le^{a} complex conformer 1	-58±4.6
*Bi*GFL-BP-Le^{a} complex conformer 2	-36±13

**Table S4: The top five structural orthologues of *Bi*GFL-BP**					
Name	Z-score	RMSD (Å)	Aligned (aa)	Total (aa)	Identity %
AcbH (PDB: 3OO6)	33.2	3.3	367	390	15
Chitooligosaccharide binding protein (PDB: 7EHP)	32.5	3.5	365	393	21
RafE SBP (PDB: 6PRE)	32.5	3.7	366	386	20
Xylobiose-BxlE (PDB: 3VXC)	32.5	3.4	370	398	16
MalE ABC transporter (PDB: 8ART)	32.5	3.4	365	393	22

This data was retrieved from a search of the DALI server (https://ekhidna2.biocenter.helsinki.fi/dali/) performed on September 24, 2024.

Table S5: Primers for cloning and mutation of *Bi*GFL-BP (Blon_0883) gene.

Cloning product	Strand	Sequence (5´→3´)
*Bi*GFL-BP wildtype	Sense	**TTTCAGGGCGCCATG**GCGGCCTGCGGTGGGGTA
	Antisense	**GACGGAGCTCGAATT**CTGCTTGGCCGCCGCGTT
*Bi*GFL-BP (insertional mutation)	Sense	**CCAGCTCAAGGGATC**TGACCACATCCAAGAAGATC
	Antisense	**CGGTACCCGGGGATC**CACGTCCTTCTTGTAGGTCA
*Bi*GFL-BP gene locus (verification of mutation)	Sense	AGTGAAGTGGCGTCCGTCGCTTC
	Antisense	TTCGATTGTGGTGTTGATGTAG
*Bi*GL-BP gene locus (verification of mutation)	Sense	GAACGGGATCTCGTGTTCCTTGTC
	Antisense	CAGAAGGACGGCTCCATCGAGATC

The bold bases in the primers are the homologous flanks for the recombination into the target plasmid.

Details of the Molecular dynamics (MD) simulations.

The references of the tools are cited in the main manuscript text, and are therefore omitted from this description, which is intended to give additional details to expert readers.

Docking and preparation of the MD systems

The MD simulations were carried out in Amber19.17. All bonds involving hydrogen atoms were frozen during the simulations with SHAKE to facilitate a larger time step of 0.002 ps. The simulations of the H1 and Le^a complexes were performed in triplicates to explore the conformational space and access statistical uncertainties. System minimizations were performed using varying numbers of steepest decent and conjugated gradient cycles to obtain different conformational starting points. This step involved 1000, 1250 and 1800 steepest descent cycles followed by 4000, 3750 and 3200 conjugated gradient minimization cycles for respectively simulation 1, simulation 2 and simulation 3. During the minimization, a 10 kcal (mol Å^2)$^{-1}$ restraint was applied on the protein backbone heavy atoms. Subsequently, the temperature of the system was raised from 10 K to 295.15K in an NVT ensemble (constant number of atoms, N, constant volume, V, and constant temperature, T). The Langevin thermostat with a collision frequency of 5.0 ps^{-1} ramped the temperature for 0.3 ns followed by a 0.7 ns simulation at the target temperature. Restraint on protein backbone heavy atoms and harmonic restraint on hydrogen bond between D210 and C4-OH (Gal) in *Bi*GFL-BP-H1 complex were applied during the heating process. Only a restrain on the protein backbone in the *Bi*GFL-BP-Le^a system was applied. Afterwards, a 0.4 ns simulation at constant pressure, p, and temperature (*NpT* ensemble) was performed to equilibrate the systems. The pressure and temperature were controlled using the Monte Carlo barostat and the Langevin dynamics with a 1.0 ps^{-1} collision frequency, respectively. The restraints were stepwise released by weaking the force constant from 500-0 kcal (mol Å^2)$^{-1}$ for the hydrogen bond and from 30-0 kcal (mol Å^2)$^{-1}$ for the backbone heavy atoms. Subsequently, constant pH MD simulations were conducted for 200 ns. During minimization and MD simulations, a cut-off of 12 Å was employed for the non-bonded interactions, while particle mesh Ewald method was employed for the electrostatic interactions. Coordinates were saved every 10 ps, and MD trajectories were analysed for ligand binding to Blon_0883.

Molecular dynamic simulations results

Magnitude of protein-ligand fluctuations

The RMSD was determined for all simulations by measuring the average change in the displacement of the backbone atoms for a particular frame, relative the first frame of the simulation. For a simulation to be considered equilibrated, the RMSD values must stabilize around a certain value,

which is used as a probe into the stability of the structural conformational changes of the protein. Thus, the changes are proportional to the conformational changes during the simulation. The RMSD values for the H1-complex rapidly stabilized around 1.5 Å for the first simulation. For simulations 2 and 3, a large increase in the RSMD was evident in the first 50 ns of the simulation. Hereafter the RMSD values fluctuate between 3-5 Å. It is apparent that RMSD values for simulations 2 and 3 show significantly larger fluctuations compared to simulation 1. However, a continued increase in the RMSD values is not seen which indicates that the structure is getting close to an equilibrated structural conformation. The RMSD values, for the first Le^a conformer fluctuate between 1 and 2 Å over the time span of the simulations, indicating a stable protein structure. For the second Le^a conformer, simulations 1 quickly reach an equilibrium structure. Although more fluctuations are observed in simulations 2 and 3, the structures stabilize after 50 ns.

Root mean square fluctuation (RMSF)

The RMSF can be useful to understand why fluctuations in the RMSD occur. The RMSF peaks indicate the areas of the protein that fluctuate the most during the simulation. The RMSF for each residue is determined by a mass-weighted average of the heavy backbone atoms. The average RMSF over all three simulations for the H1-complex and the two Le^a conformers are compared with the RMSF values for the crystal structure. RMSF for the crystal structure is determined by converting the B-factors to RMSF values and then determining the mass-weighted average for each residue.

Binding affinity

The free binding energy has been determined for all simulations using Molecular Mechanics Generalized Born Surface Area (MM-GBSA) approach in CPPTRAJ. It determines the energy difference between the bound and unbound state of the ligand-protein complexes. All energies are calculated for an ensemble of conformational snapshots where every 100th frame was extracted from the MD trajectories. Subsequently, the average over all three simulations have been determined for the complexes.
